# Simultaneous Determination of Glass Transition Temperatures of Several Polymers

**DOI:** 10.1371/journal.pone.0151454

**Published:** 2016-03-17

**Authors:** Jiang He, Wei Liu, Yao-Xiong Huang

**Affiliations:** Department of Biomedical Engineering and Key Laboratory of Biomaterials, Ji Nan University, Guang Zhou, China; University of Freiburg, GERMANY

## Abstract

**Aims:**

A simple and easy optical method is proposed for the determination of glass transition temperature (*T*_g_) of polymers.

**Methods & Results:**

*T*_g_ was determined using the technique of microsphere imaging to monitor the variation of the refractive index of polymer microsphere as a function of temperature. It was demonstrated that the method can eliminate most thermal lag and has sensitivity about six fold higher than the conventional method in *T*_g_ determination. So the determined *T*_g_ is more accurate and varies less with cooling/heating rate than that obtained by conventional methods. The most attractive character of the method is that it can simultaneously determine the *T*_g_ of several polymers in a single experiment, so it can greatly save experimental time and heating energy.

**Conclusion:**

The method is not only applicable for polymer microspheres, but also for the materials with arbitrary shapes. Therefore, it is expected to be broadly applied to different fundamental researches and practical applications of polymers.

## Introduction

The glass transition temperature (*T*_g_) is one of the most important properties that define a polymer. *T*_g_ occurs at a fairly well-defined temperature when the material ceases to be brittle and glassy in character and becomes less rigid and more rubbery[1]. Many physical properties change profoundly at the glass transition temperature, including the coefficient of thermal expansion, heat capacity, refractive index, mechanical damping, and electrical properties which are important for the applications of materials in chemistry, food, pharmacy and optics, etc.[2–5].

Numerous experimental techniques have been proposed for the determination of glass transition temperature. Differential scanning calorimetry (DSC) and differential thermal analysis (DTA) are the prevailing methods of *T*_g_ determination which measure heat capacity as a function of temperature [6,7]. However, the determined *T*_g_ value and the measuring sensitivity of the methods are strongly influenced by the sample mass and heating rate. Moreover, the measurement is a rather tedious experimental procedure. Another conventional thermal analysis technique for *T*_g_ measurement is thermo-mechanical analyzer (TMA), which measures the volume or modulus change of the sample. It is more sensitive than the methods detecting heat capacity change[8], but the result of *T*_g_ determined by this method is influenced by the size and surface roughness of the samples. In addition, an optical method based on ellipsometry can be used to measure *T*_g_ and physical aging with the inflection in a plot of refractive index versus temperature[9,10]. But this method can only measure thin or ultra-thin films; especially the result is dependent on the thickness of the film and cannot provide the bulk glass transition temperature. All the methods available currently for measuring *T*_g_ have certain limitations: there is thermal lag influencing the accuracy of *T*_g_ determination. The thermal lag depends on not only the heating/cooling rate, but also the sample mass and the thermal conductivity of both the sample and the equipment[11].

Here we describe a novel simple optical method which can easily and accurately determine the *T*_g_ of polymer samples with little thermal lag. Glass transition is a “pseudo” second order phase transition, in other words it can manifest itself as a discontinuity upon heating or cooling at a given rate of refractive index variation as a function of temperature. The refractive index of a polymer is related to its structural and physical/chemical properties as described by the Lorentz–Lorenz equation[12]:

$$\frac{n^{2}-1}{n^{2}+2}=\frac{N\rho \alpha }{3M\varepsilon }$$ (1)

Where *n* is the refractive index, *ρ* is the density, *α* is the mean polarizability, *N* is the Avogadro number, *M* is the molecular weight of polymer repeat unit, and *ε* is the vacuum permittivity. When temperature increases, thermal expansion would lead to a decrease in density, at the same time, the mean polarizability decreases while the permittivity increases[13–15]. All the three factors contribute together to a decrease of refractive index with temperature. Especially in the glass transition process, a series structural changes would occur and thus leading to greater changes in refractive index. Therefore, refractive index is more sensitive to temperature change than size parameters which were used in TMA et al. for *T*_g_ determination. For this reason, we intended to apply our microsphere imaging technique which can real-time monitor the refractive index variation with temperature for glass transition temperature determination.

The microsphere imaging technique we reported previously[16] can easily determine the RI of a polymer microsphere by simply immersing the microsphere in a liquid medium with known RI and analyzing its image. The method have several advantages for RI measurement: it can catch up very rapid RI change thus can be used for real-time monitoring of the instantaneous RI changes with time and temperature, and it can simultaneously determine the refractive indices of several microspheres at a single time. Therefore, this method is very suitable for the determination of the glass transition temperature of polymers and has the following potential benefits. First, both the size and mass of the microsphere sample (usually several ten microns in diameter) are much smaller than those used in thermal analyzing techniques, so the thermal lag of the sample could be mostly eliminated. Second, it can simultaneously determine the *T*_g_ of several polymers by monitoring a number of microspheres of different polymers at the same time to save heating energy and experimental time. To the best of our knowledge, no method available at the present is able to determine the *T*_g_ of several polymers at the same time. Third, since in the measurement the polymer microspheres are immersed in a small volume of liquid medium such as silicone oil, it is easy to heat the sample uniformly and control the heating/cooling rate while just spend little heating/cooling energy.

In this paper, we will describe the principle of the method and the experiments of using the method of microsphere imaging to measure the *T*_g_ of polymers. To demonstrate the feasibility of the method, the *T*_g_ of three kinds of polymers: Polystyrene (PS), poly (ethylene terephthalate)-glycol (PETG) and cross-linked polystyrene (CLPS) were determined. The *T*_g_ variation of polystyrene with cooling/heating rate was also tested to evaluate the influence of thermal lag in this method.

## Materials and Methods

### Principle

**Fig 1:**
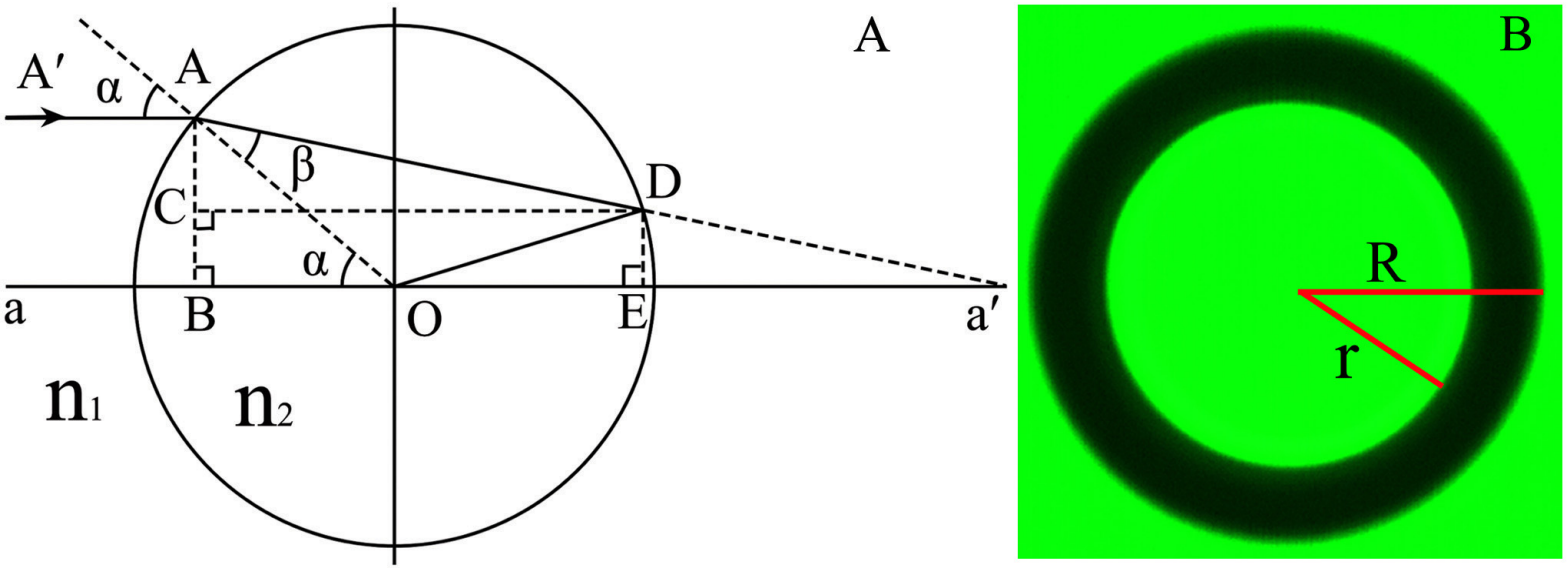
The diagram for the basic principle of RI measurement. (A) The basic principle of the method. (B) The image of a microsphere taken by a phase contrast microscope.

As described previously[16], when a microsphere is immersed in a liquid medium and illuminated by a parallel light propagating along its optical axis, a dark ring appears in the image of the microsphere if the refractive index of the microsphere *n*_2_ is greater than the refractive index of the surrounding medium *n*_1_ (see Fig 1). Let *k* = *n*_1_/*n*_2_, *DE* (the distance from point D to the axis a-a’) can be obtained by:

$$DE=2Rk^{2}{\text{sin}}^{3}\alpha -R\text{sin}\alpha +Rk\sqrt{1-k^{2}{\text{sin}}^{2}\alpha }\text{sin}2\alpha$$ (2)

And the maximum value of *DE* (*DE*_max_) generated by any parallel paraxial incident rays is just the radius (*r*) of the central bright spot in the image of the sphere shown in Fig 1B. *DE*_max_ can be obtained by setting $\frac{\partial (DE)}{\partial \alpha }$ = 0 and then the following equation can be derived: [16]

$$n_{2}=n_{1}{\left(\frac{3}{8}\sqrt[{3}]{X^{2}+\sqrt{X^{4}-X^{6}}}+\frac{3X^{2}}{8\sqrt[{3}]{X^{2}+\sqrt{X^{4}-X^{6}}}}+\frac{1}{2}\right)}^{-1/2}$$ (3)

Therefore, by using a medium with known refractive index *n*_1_ and measuring the ratio *X* (*r*/*R*) from the image of the microsphere, one can easily determine the refractive index *n*_2_ of the microsphere with Eq (3).

### Setup of the measurement

**Fig 2:**
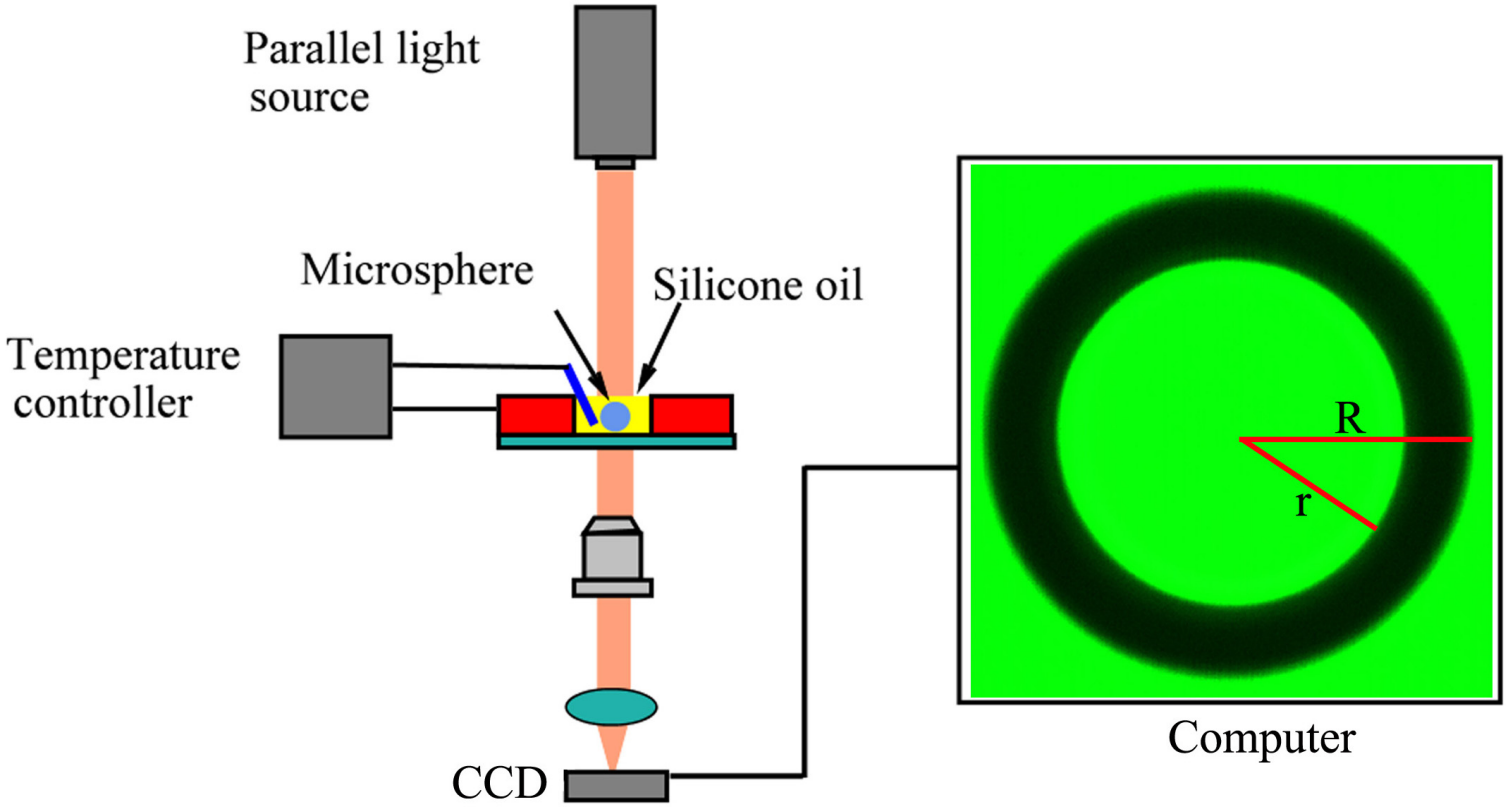
The schematic diagram of the real-time microsphere imaging system.

We developed a specific experimental setup for *T*_g_ measurement as shown in Fig 2. The setup consists of a parallel light source, a micro-imaging system which used a CCD digital camera via a 40× objective for imaging, and a small glass dish with temperature controller. The parallel light from a 532 nm LED light source was incident to the microsphere immersed in silicone oil in the glass dish. The image of the microsphere was then taken automatically at each selected temperature with the micro-imaging system which was controlled by a homemade image processing software for auto-focusing and target identification. The temperature of the microsphere and silicone oil was maintained by the temperature controller. The variation of temperature was controlled to within ± 0.1°C.

The image processing software was also responsible for the intelligent image target identification and analyzing. During the target identification and analyzing process, both *R* and *r* were determined automatically by finding the maximum gradient values at the edges of both the central bright spot and the outer radius of the microsphere using the method of super-resolution restoration analysis. The standard deviation of the *R* and *r* measurement is estimated to be 50 nm when using a 5M pixels camera for imaging, this corresponds to a standard deviation of 0.00015 in RI determination.

### Preparation of polymer microspheres

Polystyrene (PS) was purchased from Aldrich, USA, average Mw = 230k g/mol; poly (ethylene terephthalate)-glycol (PETG) was purchased from Eastman Chemical Co., USA, average Mw = 200k g/mol; and the styrene for cross-linked polystyrene (CLPS) were purchased from Tianjin Guangfu Fine Chemical Research Institute, China. PS microspheres and PETG microspheres were prepared using the emulsion solvent removal method[17]. Atactic PS (1.2g)/PETG(0.8g) were dissolved in chloroform(20ml) as oil phase, and PVA(1g) dispersed in distilled water(150ml) as aqueous phase, the oil solution was slowly added dropwise to the aqueous solution with magnetic stirring at 1000/800 rpm. The beaker was tightly sealed with aluminum foil to prevent evaporation of chloroform during the emulsification-diffusion process for 5 h. Afterwards the microspheres were collected by centrifugation and washed before drying. PVA(1750±50) were purchased from ZhanCheng Company. The CLPS microspheres were synthesized by dispersion polymerization[18]. Analytical graded Styrene (70% of total amount), AIBN (initiator, from PuBo Company), DVB(crosslink, from PuBo Company), PVP(stabilizer, from PuBo Company) and ethanol(solvent, from PuBo Company) were introduced into a reaction vessel, and heated to 70°C, stirred at 120rpm for 24h. Nitrogen gas was bubbled through the solution for deoxygenation. The microspheres were then collected by centrifugation and washed before drying.

### Measurement on the local temperature with silica microsphere as the sensor

Since the thermos-optical coefficients of some solids such as silica are very small(-10×10^−6^°C)[19] in comparison with that of liquids, their refractive indices can be approximated as a constant in a range of temperature(such as 20°C to 130°C). Therefore, silica microsphere can act as a sensitive sensor for the determination of local temperature in a solution. As the thermo-optical coefficient for most liquids is about—4.0×10^−4^°C, the precision of the temperature measurement using silica microsphere as a temperature sensor is about 0.4°C with the setup using in our experiment. The advantage of using silica microspheres as a temperature sensor is that they can be placed closely to the polymer microsphere samples under glass transition temperature measurement, so that real-time local temperature variation of the polymer sample can be determined directly and accurately.

### Measurement on the RI of silicone oil as a function of temperature

Silicone oil is a common solvent used in polymer thermal analysis for it has good thermal conductivity and linear thermo-optical coefficient. In our experiment, we used silicone oil (dimethyl silicone oil, M_n_ ≈ 6000, viscosity = 100 cs, purchased from Aladdin, China) as the immersion medium. Prior to test the polymer microsphere samples, the RI of silicone oil as a function of temperature was calibrated using a silica microsphere sensor as described in the last section.

It should be noted that though silicone oil is a good solvent and frequently used in polymer thermal analysis, it is not the only choice for our method. Besides the consideration of good thermal conductivity and linear thermo-optical coefficient, one should use solvents which are inert to the polymer under study as the immersion medium to prevent chemical alternations in the polymer induced by the solvent.

### Measurements on the RI of polymer microspheres as a function of temperature

The polymer microsphere samples were immersed in silicone oil. They were annealed at a temperature 130°C (above *T*_g_) for 10 min and then cooled down from 130 to 30°C with a cooling rate of 10°C/min. At the same time their images were taken at selected temperature. The *r* and *R* values of the spheres’ images were measured, thereafter the ratio of *r* to *R* was used to calculate the refractive index of the microspheres using Eq (3) with the refractive index of the silicone oil at the temperature.

### Measurements on the effect of cooling rate on the glass transition temperature

The glass transition temperature of PS was observed at seven different heating rates (0.2, 0.5, 1, 2, 5, 10, 20°C /min) to determine the dependence of the glass transition temperature on cooling rate.

### Statistical analyses

In the experiment, at least six microspheres were measured for the *T*_g_ determination of each polymer. The mean and standard deviation of all the data were obtained by performing statistical analyses with the SPSS 15.0 statistical software (IBM, USA). The typical standard deviation in the RI measurements was about 0.0005.

## Results

**Fig 3:**
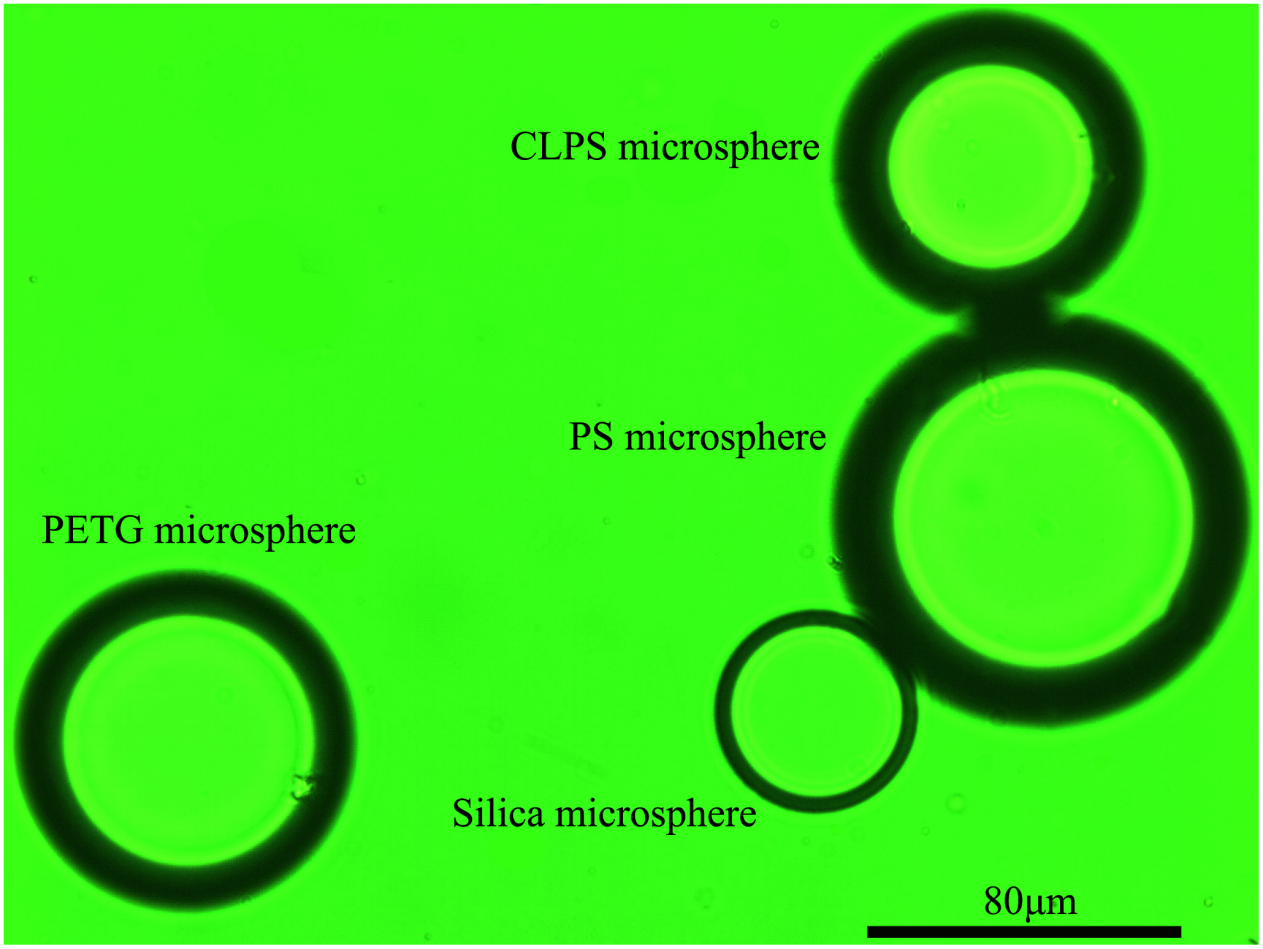
The images of the three kinds of polymer microspheres. The microspheres were immersed in silicone oil at 25°C accompanied with a silica microsphere as the temperature sensor.

**Fig 4:**
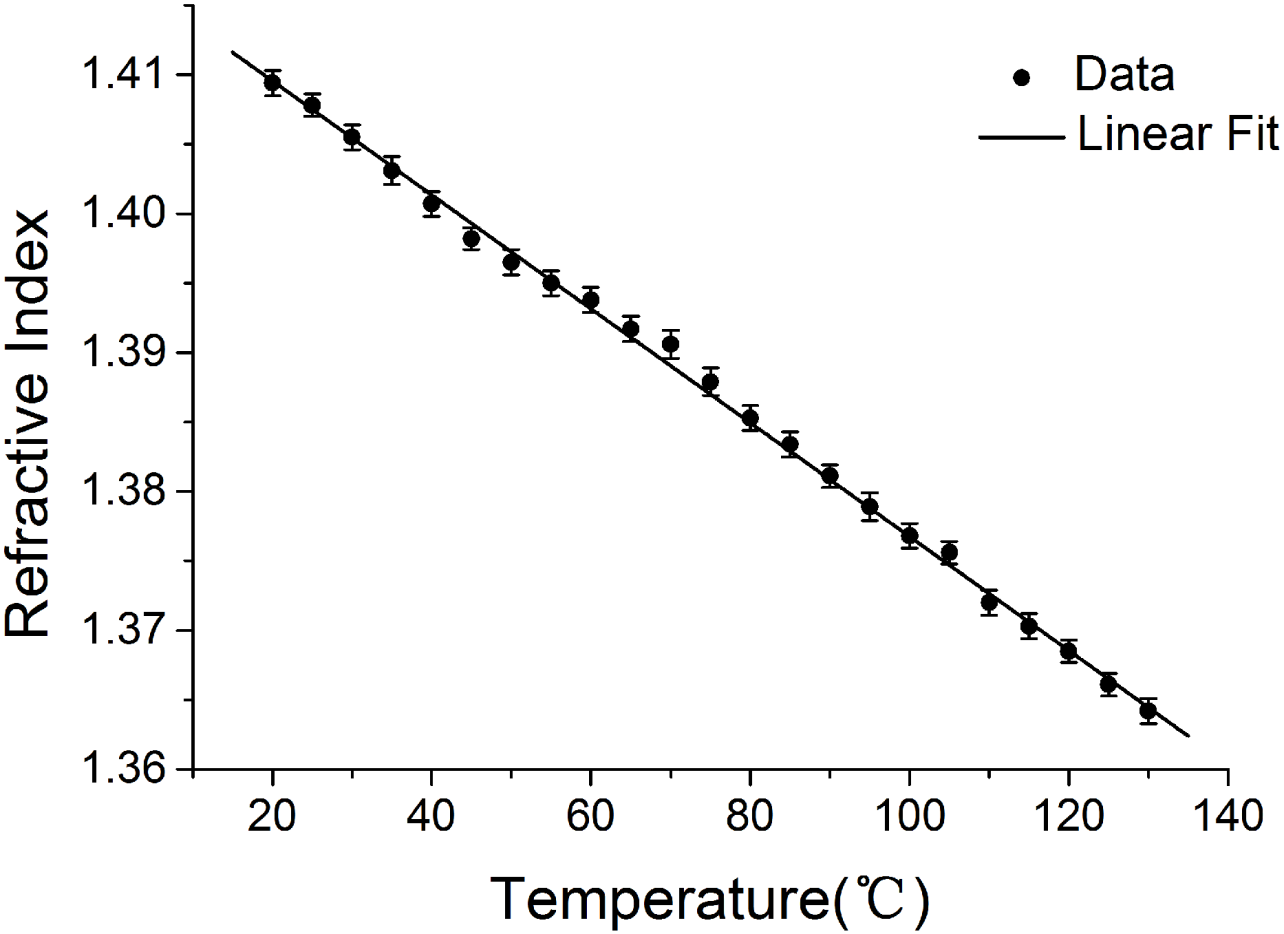
The refractive index of silicone oil vs. temperature.

Microspheres of different sizes were prepared for each kind of the polymers. Fig 3 shows the image of the three kinds of polymer microspheres immersed together in silicone oil in a measurement accompanied with a silica microsphere which was acting as a temperature sensor. Fig 4 illustrates how the RI of the silicone oil purchased from Dimethyl (100cs) changes with temperature from 20°C to 130°C. We can see that the RI value of the oil changes linearly with temperature and follows the relation of *n*_1_ = 1.4094 + 3.9×10^−4^ × [T(°C)– 20], indicating that it is an ideal immersion medium for the method.

**Fig 5:**
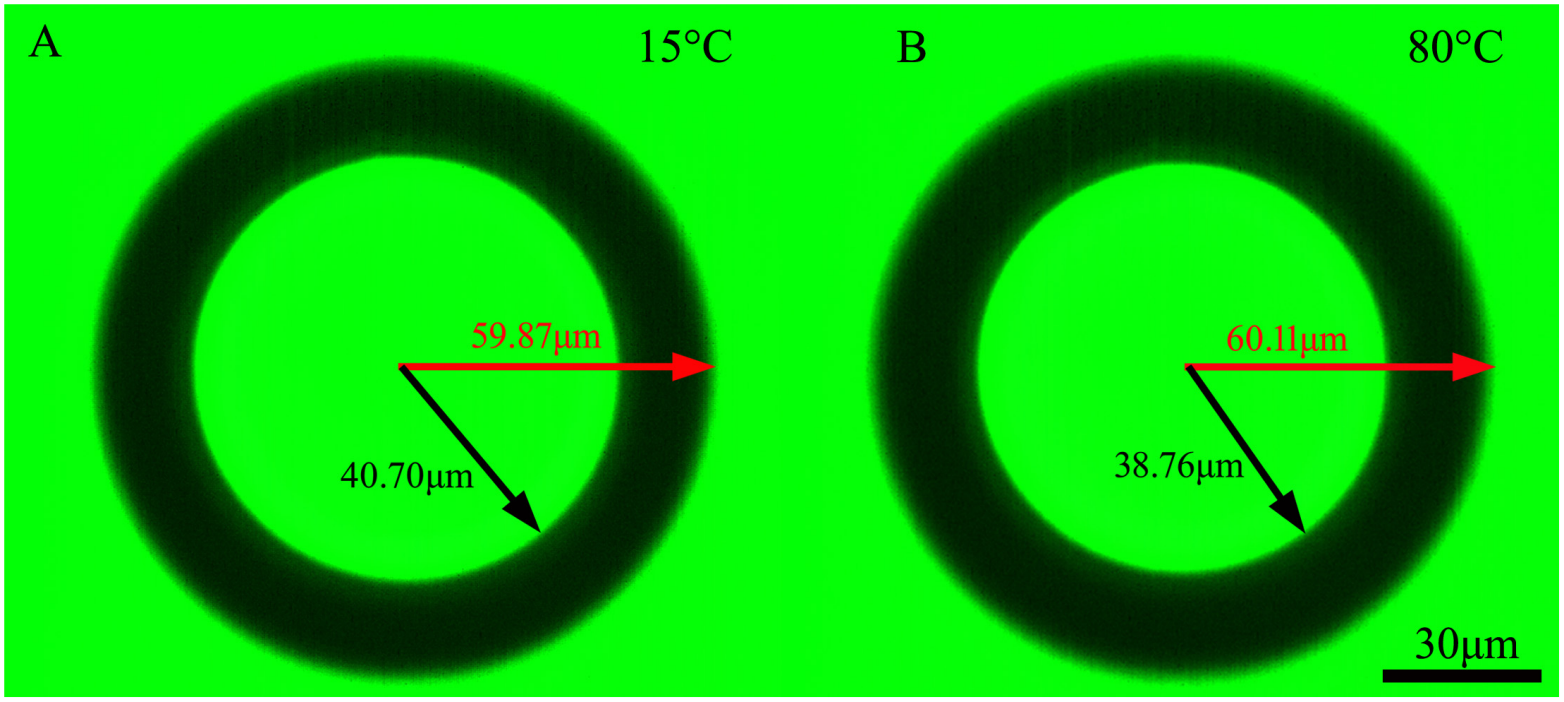
The images of a PS microsphere taken at 15°C and 80°C respectively.

Fig 5 is the images of one of the microspheres (PS) taken at 15°C and 80°C respectively. We can see that, as temperature rose, *R* increased whereas *r* decreased. By using the *R* and *r* values of each microsphere at every temperature, and the RI value of the silicone oil shown in Fig 4 at every temperature, the refractive indices of the microspheres were calculated using Eq (3).

**Fig 6:**
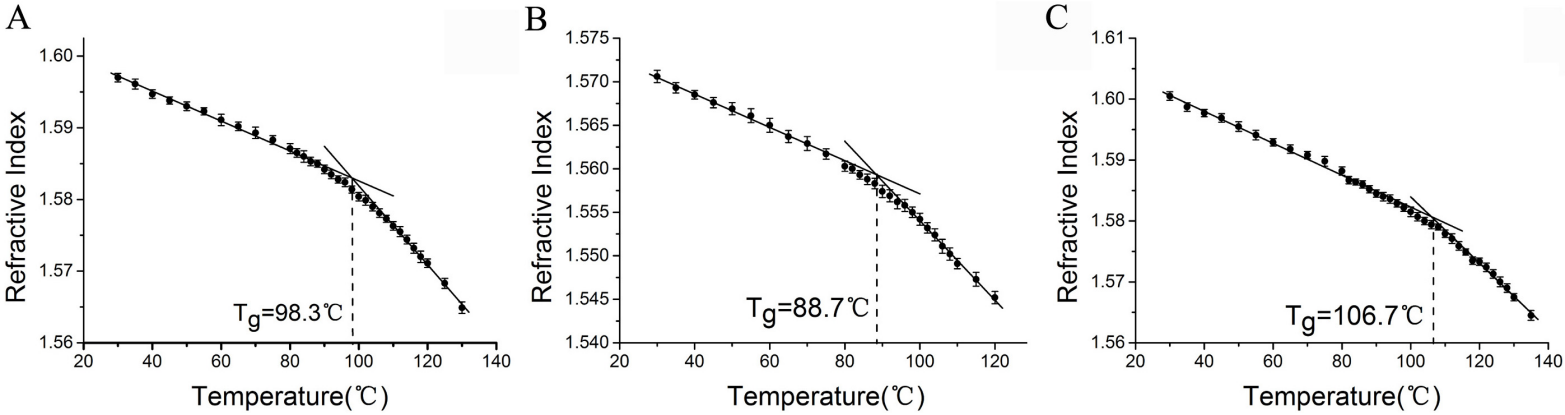
The refractive indices of different microspheres vs. temperature. (A) Polystyrene, (B) Poly (ethylene terephthalate)-glycol, (C) Cross-linked polystyrene.

Fig 6 shows the refractive indices of the three polymers versus temperature respectively. We can see that in all the polymers, RI decreases linearly from 35°C with increasing temperature, but change its slope after passing a temperature region. The range can be distinguished into three regions: the glassy (low-T) and rubbery (high-T) regions separated by an intermediate transition region. *T*_g_ was defined as the intersection point of the two best-fit linear lines. We can see that the *T*_g_ of PS and PETG microspheres are about 98.3°C and 88.7°C, quite consistent with the results determined by DSC for the materials with similar molecular weights [20,21]. The *T*_g_ of CLPS is about 106.7°C, which is also consistent with the results reported in the literature [22]. Since the standard deviation of the determined refractive index in the measurement was estimated to be 0.00015, and the slope of the curve in rubbery region is steeper than that in glassy region, so the temperature uncertainty in glassy region is about 1°C but 0.3°C in rubbery region.

**Fig 7:**
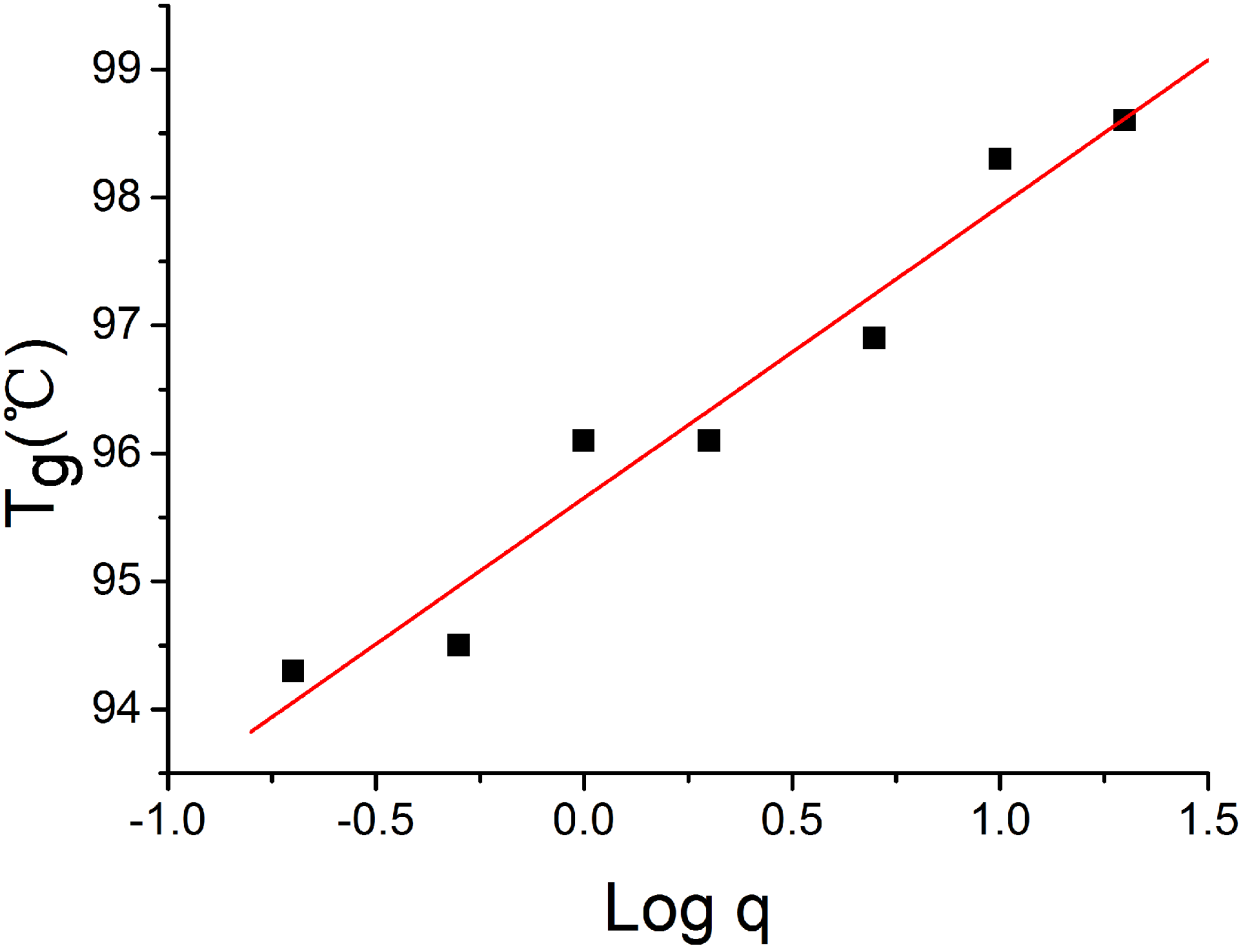
Glass transition temperature (*T*_g_) as a function of cooling rate *q* (°C/min) for PS.

Fig 7 shows *T*_g_ as a function of cooling rate *q* (°C/min) for PS. As expected, the glass transition temperature increases with cooling rate. It changes about 2.0°C / log *q* that is less than the result obtained by DSC [23].

## Discussion

The process of glass transition is a relaxation process of polymer material, so *T*_g_ value is dependent on the testing conditions including the cooling/heating rate and the size of polymer as there is thermal lag between the heat conducting medium and the sample and between the center of the sample and its surface[24]. To reduce thermal lag, we chose silicone oil as the liquid medium for it has good thermal conductivity. Since in the experiment, very small volume of silicone oil was used to just immerse the microspheres, so it is convenient to change and control the oil’s temperature and conduct heat to the immersed microspheres. On the other hand, thermal lag also depend on the size and shape of the sample, due to the low thermal conductivity of common polymers, large sample size will increase the temperature lag, so the *T*_g_ determined by DSC has a greater variation with cooling/heating rate in which larger size sample is used and the samples are usually not symmetry in shape. However, in our method, the size of the polymer microspheres are as small as several ten microns, and the spheres are symmetry in shape, therefore heat can be quickly diffused from all directions of the immersing oil into the whole sphere uniformly so that the sphere can become thermal equilibrium faster at each desired temperature. Consequently, most thermal lag can be eliminated thus the *T*_g_ value determined by the present method could be more accurate.

There are some other factors also affecting the *T*_g_ variation with cooling/heating rate. As described in the Introduction section, density, the mean polarizability and the permittivity contribute together to the variation of refractive index when temperature is changed, so refractive index is more sensitive to temperature than volume does, and its variation can follow up temperature change faster than volume change. From Fig 5 we can see that, when temperature rose from 15°C to 80°C, both *R* and *r* changed. However, the change of *r* (Δ*r* = 1.94 μm) was much greater than the increment in *R* (Δ*R* = 0.31 μm). As we know that the increment in *R* corresponds to the volume thermal expansion of the microsphere, while according to Eq (3), the change in *r* is due to the refractive index variation of the sphere for a certain microsphere and surrounding medium. So the sensitivity of measuring the refractive index variation with temperature is about six fold as that measuring volume change. This finding is not only significant for glass transition temperature determination, but would be also meaningful in many optical applications.

On the other hand, in DSC and DTA measurements, the temperature lag between the sample and the thermoelectric probe for temperature detection, and even the placement of the thermocouple would be also factors affecting the accuracy of *T*_g_ determination. In contrast, our method used a silica microsphere temperature sensor which was placed closely to the sample under measurement (see Fig 3), so the local temperature variation of the sample can be followed up quickly and detected more accurately.

Unlike the conventional methods which can only test one polymer sample at a time, our method can simultaneously determine the glass transition temperatures for several microspheres of different polymers in a single experiment. As shown in Fig 3, by immersing the PS microsphere, the PETG microsphere and the CLPS microsphere together in silicone oil under the same field of view, we determined the glass transition temperatures of the three polymers in a single experiment. The power consumption of our method for a *T*_g_ determination experiment was about 200 W, it is much lower than that in DSC and DTA (typically about several KW). Therefore, compared with the conventional methods by which each experiment can only detect one polymer, our method can greatly save experimental time and cooling/heating energy when determining the *T*_g_ of several polymers in one experiment. The way to do so is simple and just capturing the images of the microspheres together at each temperature, and then analyzing their images with Eq (3) afterwards respectively for RI determination.

**Fig 8:**
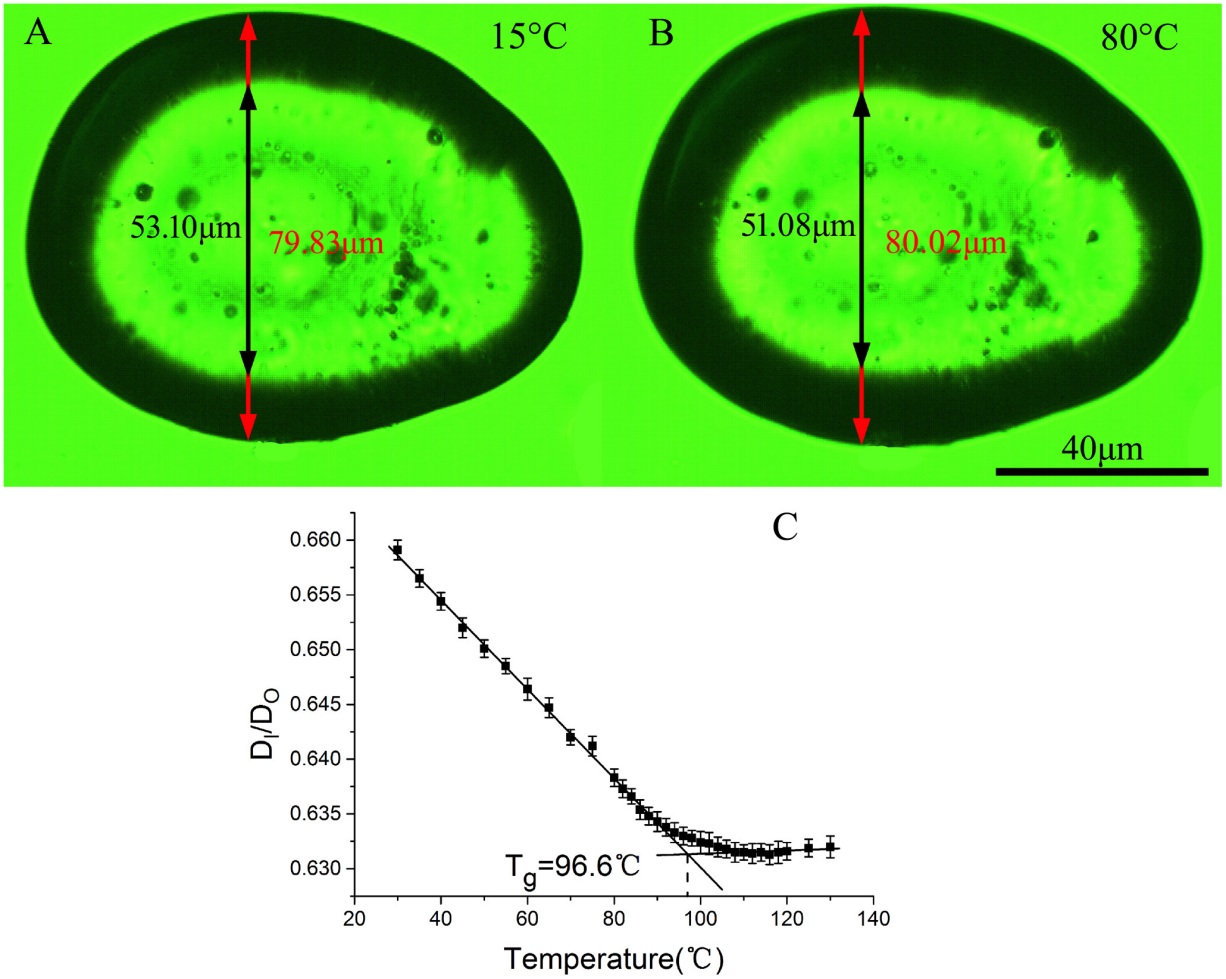
The measurement of a slice of PS. The images of a slice of PS with arbitrary shape taken at (A) 15°C and (B) 80°C respectively, and (C) is D_I_/D_o_ as a function of temperature.

For those materials which have difficulty in microsphere preparation, our method is also applicable. Fig 8 shows the images of a slice of PS with arbitrary shape at two different temperatures, we can see that both of them have dark rings since the effect of refraction. Similar to the case in microsphere imaging, the dark ring of the slice becomes wider as temperature increases, indicating that the ratio between the refractive index of the PS (*n*_2_) and the refractive index of its surrounding medium (*n*_1_) becomes smaller. Though it doesn’t like the case of microsphere imaging in which a defined relationship between *n*_2_ and *r*/*R* is known(see Eq (3)) for the calculation of the absolute value of *n*_2_, one can still obtain the information about the RI variation with temperature from the changes of both D_I_ and D_o_ (see Fig 8A and 8B). The glass transition temperature therefore can be still estimated. Similar to the method of microsphere imaging in which *n*_2_ is a function of the ratio *X* = *r*/*R* (see Eq (3)), we also used the variation of the D_I_/D_o_ ratio with temperature to determine *T*_g_. Fig 8C shows D_I_/D_o_ as a function of temperature for the slice of PS shown in Fig 8A, which was immersed in silicon oil of 130°C for 10 minutes and then cooled down from 130°C to 30°C with a cooling rate of 10°C/min. We can see that D_I_/D_o_ decreases with temperature linearly but change its slope after passing the glass transition temperature region, so *T*_g_ can be also determined from the intersection point of the two best-fit linear lines. The *T*_g_ obtained in this way (96.6°C) is a little bit smaller than that obtained by microsphere imaging measurement (98.3°C), but still within the range of the literature results[20,21,25]. Nevertheless, in the case of microsphere imaging, the refractive index of the PS sample can be accurately calculated using Eq (3), so *T*_g_ would be obtained with better accuracy.

The limitation of our method is that the temperature range for the measurement is limited by the liquid medium used for immersing the sample, so it would be narrower compared to DSC, etc. Furthermore, the microsphere preparation also takes time in sample preparation. Though one can just use a slice of sample with arbitrary shape for the measurement as described in the last paragraph, the *T*_g_ so determined probably is not so accurate as that using microsphere imaging for the determination.

## Conclusions

We have developed a simple and easy method which can monitor the refractive index variation of several polymer microspheres with temperature together so that the glass transition temperatures of different polymers can be determined simultaneously in a single experiment. It was demonstrated that by measuring the refractive index of very small microspheres changing with temperature, the present method has sensitivity about six fold better than the conventional methods and can eliminate most thermal lag at the same time. So the method can measure the glass transition temperature with better accuracy and the determined *T*_g_ has less variation with cooling/heating rate. The method is not only applicable for microspheres, but can be extended to the materials with arbitrary shapes. With the advantages of better sensitivity and accuracy, simple and easy in performance while saving time and energy, the method is expected to be broadly applied to both fundamental researches and practical applications of polymers. Based on the experimental setup we developed specially for the measurement, it is possible to develop it into a powerful but simple instrument for the measurements of *T*_g_ and some other properties of polymers such as refractive index and thermal expansion.
